# Supplementary material for: TLR7 controls myeloid-derived suppressor cells expansion and function in the lung of C57BL6 mice infected with Schistosoma japonicum
Source: PLoS Negl Trop Dis. 2022 Oct 24;16(10):e0010851. doi: 10.1371/journal.pntd.0010851 (PMC9591064; doi:10.1371/journal.pntd.0010851)
Supplement: Attachment — Submitted filename: Response to reviewers.pdf [file pntd.0010851.s002.pdf]

Response:
Manuscript (ID: PNTD-D-22-00828)

We thank the reviewers for these positive comments on the work. We really appreciate your constructive suggestions, which help improve the quality of our paper. We have made point-by-point responses to your comments below and all given suggestions were considered and incorporated in the revised manuscript. ***All changes have been highlighted in the revised manuscript for the easy of reading.***

Methods

Reviewer #1: The objectives are clearly defined, and the design is appropriate, however, more details should be given in Material and methods, it is not given even the number of mice used in the study. Some methods, ie how the area of granuloma was calculated (how it was measured, number of granuloma measured in each mouse…) is not given in material and methods but it is mentioned in results and in figure 6E. Some details should also be added to the statistical analyses, ie. if the data were parametric or not. This is important to know the appropriate test to apply. The ethical issues are satisfactorily addressed.

Response: *Your questions are very important. We have followed your suggestion, and more details have been added to the revised "Materials and methods", including the source of reagents (company, product code) in "Reagents and antibodies", the method of calculating the area of granuloma in "Histology studies" and the details of the statistical analyses in "Statistics" and "Figure legends". In addition, the number of mice was added in the legend and the sample size in this study was referred to in the "Supporting Information". Thank you!*

Reviewer #2:

1. In Reagents and antibodies, please specify the source of reagents (company, product code).

Response: *Thank you for your suggestion, and the source of reagents (company, product code) used in this study were added in the revised "Materials and methods".*

2. In Parasites and infection, "C57BL/6 mice were infected percutaneously with 40 ± 5 cercariae". In figure1, "lymphocytes were isolated from *S. japonicum* infected mouse lung. FACS was used to sort MDSCs, which were then co-cultured with ConA pre-stimulated, CFSE-stained T cells from BALB/c mice at a 1:2 ratio", Why are MDSCs derived from C57BL/6 mice and T cells from BALB/c mice?

Response: *That's a good question. Because of the difference in D and DP antigens in HLA class II antigens, T cells from two unrelated individuals with normal function can be stimulated each other to proliferate in vitro, which is known as mixed lymphocyte culture. The greater the degree of HLA antigen difference between two individuals, the stronger the response. Therefore, MDSCs derived from C57BL/6 mice and T cells derived from BALB/c mice can more accurately reflect the inhibitory function of MDSCs. The protocol is based on the previous reports (Dehong Y et al. Eur J Immunol. 2013; Quan Y et al. J Immunol. 2017).*

Results

Reviewer #1: The results match with the study design and objectives and they are clearly presented, but there are some concerns about the terminology used in histological pictures. Thus,

in figure 1B thus authors state that downward arrow indicates interstitial dilation, however this dilation is rounded and similar to that observed in the picture of the control group. These dilations are consistent with central lobular veins, an interstitial dilation due to a lesion usually is irregular in shape, in any case higher resolution should be required for an appropriate identification of these structures. I recommend to delete “and the downward arrow indicates interstitial dilation”.
Response: *Thank you. We have followed your suggestion and removed the phrase "and the downward arrow indicates interstitial dilation" from the revised manuscript. (please see updated Fig. 1B).*

In Figure 6D the authors state that upward arrow indicates interstitial dilation, this arrow points an area of interalveolar septal thickening, thus the appropriate terminology here is “interstitial thickening”. In addition, the authors state that the right arrow indicates alveolar thickening while this arrow indicates “alveolar dilation”.
Response: *You are right. Thank you for your suggestion, and it has been corrected in the revised manuscript.*

Since granulomas are typical lesions in schistosomiasis, even the author presents the area of granulomas in Fig. 6E, it would be more interesting for readers a figure showing lung granulomas in both groups instead of a picture showing alveolar dilation and interstitial thickening.
Otherwise the remaining tables and figures are of good quality.
Response: *Thank you for your suggestion, and the lung granulomas in both groups were added in the revised manuscript (please see updated Fig. 6D).*

Reviewer #2: (No Response)

Conclusions
Reviewer #1: Discussion is appropriate and includes recent references on the field of the research. However, conclusions are very brief and the authors have not included conclusions for some of their relevant results, for example the role of TLR7 in pulmonary lesions caused by S. japjonicum.
Response: *Thank you for your suggestion. MDSCs have been described as $Gr1^{+}CD11b^{+}$ immature myeloid cell populations derived from monocytes and polymorphonuclear granulocytes that migrate from the blood to the site of infection (Onyilagha C et al. J. Immunol, 2018; Veglia F et al. Nature Reviews. Immunology, 2021). MDSCs are recruited to the site of inflammation in several models of inflammation (Valanparambil R M et al. PLoS Pathogens, 2017; Tcyganov EN et al. J Clin Invest, 2021; Haverkamp JM et al. Eur J Immunol. 2011). In this study, TLR7-KO could dramatically increase the percentage of MDSCs infected with S. japonicum (Fig 3E) and the lungs of infected TLR7-KO mice showed more infiltration of inflammatory cells, interstitial dilation and granuloma compared with infected WT mice (Fig 6D and 6E), which suggested that TLR7 could inhibit the pulmonary inflammation caused by S. japonicum infection. Consistent with us, TLR7-KO mice accumulate increased numbers of pulmonary $Gr1^{+}CD11b^{+}$ MDSCs and show a more intense inflammation in the lung tissues during IAV infection compared to wild type mice (Jeisy-Scott V et al. PloS One, 2011). On the other hand, our previous studies also showed that TLR7 knockdown caused serious splenomegaly in infected mice (Wei H et al. PLoS Negl Trop Dis, 2021). It suggested TLR7 could inhibit the inflammation response in the spleens of S. japonicum*

infected mice. TLR7 signaling has been reported to orchestrate inflammation and innate immunity upon EV71 infection, which is consistent with our findings (Luo Z et al. Plos Pathogens, 2017).

Furthermore, It has been previously shown that the presence of MDSCs skews the immune response towards a Th2 response (Delano MJ et al. J Exp Med, 2007; Sinha P et al. J Immunol, 2007; Jeisy-Scott V et al. PloS One, 2011). This is mainly attributed to their ability to induce IL-10 and reactive oxygen species (J Immunol, 2007; Jeisy-Scott V et al. PloS One, 2011; Cesar A Corzo et al. J Immunol, 2009). TLR7-KO MDSCs produced IL-10 as well as IL6 in mice lung after S. japonicum infection (Fig 4C). Interestingly, IL6 has been shown to amplify the Th2 response (Tibbitt CAet al. Immunity. 2019). The ability of TLR7-KO MDSCs to coproduce IL-10 and IL6 may be one mechanism that encourages the Th2 bias observed in TLR7-KO mice during S. japonicum infection. It suggested that TLR7 deficiency might increase MDSCs accumulation and Th2 biased response to S. japonicum infection in the lungs of mice, which might be related to pulmonary lesions in infected TLR7-KO mice (eisy-Scott V et al. PloS One, 2011; Leist S R et al. Cell, 2020).

Moreover, the mechanism of immune response induced by schistosoma japonicum is very complex: non-specific immunity and specific immunity are mutually conditional and complementary; humoral and cellular immunity regulate and balance each other; interaction between schistosoma antigens and host MHC; a variety of immune cells, including macrophages, NK cells, B cells and T cells, are involved in the pulmonary lesions in schistosomiasis, and these factors may affect the pathogenesis of schistosomiasis (Zhao Y et al. BMC Infect Dis. 2019; Houlder EL et al. Front Immunol, 2021; Souza COS et al. Front Immunol, 2018). Therefore, in the lungs of infected mice, TLR7-KO may affect the immune response of other immune cells, such as B cells and NK cells, and jointly affect the pulmonary lesions of infected mice (Wei H et al. PLoS Negl Trop Dis, 2021; Dianhui Ch et al. Innate Immun, 2019). This needs to be further studied. We included it in the revised "Discussion".

Reviewer #2: (No Response)

Editorial and Data Presentation Modifications?

Reviewer #1: The manuscript should be revised for typographical errors, some of them are:
-line 64: add space after schistosomiasis
-line 72: add space after animals
-line 380: delete space after (Fig 3B)
-line 407: S. japonicum should be in italic
-line 435: delete space after (Fig 6D and 6E)
-line 536: delete space after Md
-line 575: delete space after Immunotherapy
-line 621: delete space after shown
-line 665: delete comma after of and delete space before and after /
Response: *Thanks, the errors were corrected in the revised manuscript.*

Reviewer #2: (No Response)

Summary and General Comments

Reviewer #1: The manuscript describes the characteristics of MDSCs in the lung of *S. japonicum* infected C57BL/6 mice, and the role of TLR7 on the progression of MDSCs activation and differentiation in the lung of *S. japonicum* infected mice. The work is original and presents new and interesting information to better understand the pathogenesis of schistosomiasis. In general, the paper is well designed and well written, but some more details should be given in material and methods.

Response: *Thank you for highlighting this important point. We have followed your suggestion, and more details have been added to the revised "Materials and methods", including the source of reagents (company, product code) in "Reagents and antibodies", the method of calculating the area of granuloma in "Histology studies" and the details of the statistical analyses in "Statistics" and "Figure legends". In addition, the number of mice was added in the legend and the sample size in this study was referred to in the "Supporting Information". Thank you!*

Reviewer #2: The manuscript entitled "TLR7 controls myeloid-derived suppressor cells expansion and function in the lung of C57BL6 mice infected with Schistosoma japonicum" by Lu Zhou et al. This study illustrated that TLR7 could delay the progression of *S. japonicum* infection-induced lung disease mainly through MDSCs. TLR7 deficiency aggravates *S. japonicum* infection-induced damage in the lung, with more inflammatory cells infiltration, interstitial dilatation and granuloma in the tissue. However, some results or some description are confusing.

1. Why did the authors choose the sixth week of schistosome infection to observe MDSCs in lung? What are the potential implications?

Response: *Usually, S. japonicum began to lay eggs at 3-4 weeks after infection. On week 6-7 the immune response of MDSCs will reach to a peak (Fig 2), which is consistent with the previous report (Quan Yang et al. J. Immunol. 2017), and mouse will begin to die on week 7. So, the sixth week post infection was chosen as the time point in this study. Thank you!*

2. Why did the author investigate TLR7? TLR7 is an endosomal TLR that recognizes single-stranded RNA (ssRNA) and mediates early innate immune responses to viruses, bacteria and malaria. Recently, TLR7 agonists were found to be therapeutics against viral infections and bacteria. SEA and R848 were added to the cells alone or together, and a negative control was used as described in Materials and Methods. Why add soluble egg antigen instead of ssRNA to culture system?

Response: *Thank you for your suggestion. Many kinds of TLRs were found to be expressed on MDSCs during the immune response induced by invaded pathogens (Jeisy-Scott V et al. PloS One, 2011; Ray A et al. Front Cell Infect Microbiol. 2013). To explore the effects of TLRs on pulmonary MDSCs in the course of S. japonicum infection, we compared the expression of TLR2, TLR3, TLR4 and TLR7 in $CD11b^{+}Gr1^{+}$ cells sorting from naive and infected mice lung. Among these TLRs, the expression of TLR7 increased significantly in pulmonary $CD11b^{+}Gr1^{+}$ MDSCs, while the expression of TLR2, TLR3 and TLR4 showed no significant difference after infection (Fig 3A). Furthermore, compared with the expression of TLR7 in $CD11b^{+}Gr1^{-}$ cells and $CD11b^{-}Gr1^{-}$ cells, the expression of TLR7 was highest in $Gr1^{+}CD11b^{+}$ cells from naive mice lung (Fig 3B). It suggested that TLR7 might be the main factor regulating the accumulation of MDSCs in the course of S. japonicum infection, that is why we investigated TLR7 on pulmonary MDSCs. Please*

refer to the updated "Discussion".

TLR7 is an endosomal TLR that recognizes single-stranded RNA (ssRNA). The reason of SEA instead of ssRNA to culture system in our study maybe as follows: first of all, in our study, MDSCs could respond to SEA via TLR7 (Fig 3D and 5C). Secondly, SEA released by mature eggs in the body is a mixture. The mixture is likely to contain substances such as exosomes, which contain complex RNA and proteins. In fact, our SEA was extracted from the in vitro lysis of Schistosome eggs, which is a crude extract mixture. It is inevitable to have single-stranded and double-stranded RNA inside. Nonetheless, the effect of ssRNA from S. japonicum on pulmonary MDSCs needs to be further investigated. We included it in the revised "Discussion".

3. TLR7 deficiency can significantly increase the percentage of PD-L1 or PD-L2- expressed MDSCs in mouse lung after infection (Fig 4B), which indicated that TLR7 deficiency promoted the immunosuppressive function of *S. japonicum* infection- induced MDSCs by up-regulating the expression of PD-L1 or PD-L2 in mouse lung after infection. However, the results indicated that the lungs of infected TLR7-KO mice showed more infiltration of inflammatory cells, interstitial dilation and granuloma compared with infected WT mice (Fig 6D and 6E), which suggested that TLR7 deficiency might aggravates lung damage in the course of *S. japonicum* infection. The results seem contradictory.

Response: *Thank you. This is a very insightful suggestion. In this study, Although TLR7 deficiency can significantly increase the percentage of PD-L1 or PD-L2-expressed MDSCs in mouse lung after infection (Fig 4B), which indicated that TLR7 deficiency promoted the immunosuppressive function of S. japonicum infection-induced MDSCs by up-regulating the expression of PD-L1 or PD-L2 in mouse lung after infection. However, MDSCs have been described as* $Gr1^{+}CD11b^{+}$ *immature myeloid cell populations derived from monocytes and polymorphonuclear granulocytes that migrate from the blood to the site of infection (Onyilagha C et al. J. Immunol, 2018; Veglia F et al. Nature Reviews. Immunology, 2021). MDSCs are recruited to the site of inflammation in several models of inflammation (Valanparambil R M et al. PLoS Pathogens, 2017; Tcyganov EN et al. J Clin Invest, 2021; Haverkamp JM et al. Eur J Immunol. 2011). In this study, TLR7-KO could dramatically increase the percentage of MDSCs infected with S. japonicum (Fig 3E) and the lungs of infected TLR7-KO mice showed more infiltration of inflammatory cells, interstitial dilation and granuloma compared with infected WT mice (Fig 6D and 6E), indicating that TLR7 could inhibit the pulmonary inflammation caused by S. japonicum infection. Consistent with us, TLR7-KO mice accumulate increased numbers of pulmonary* $Gr1^{+}CD11b^{+}$ *MDSCs and show a more intense inflammation in the lung tissues during IAV infection compared to wild type mice (Jeisy-Scott V et al. PloS One, 2011). On the other hand, TLR7 knockdown caused serious splenomegaly in infected mice, according to our previous studies (Wei H et al. PLoS Negl Trop Dis, 2021). It suggested TLR7 could inhibit the inflammation response in the spleens of S. japonicum infected mice. TLR7 signaling has been reported to orchestrate inflammation and innate immunity upon EV71 infection, which is consistent with our findings (Luo Z et al. Plos Pathogens, 2017).*

Furthermore, It has been previously shown that the presence of MDSCs skews the immune response towards a Th2 response (Delano MJ et al. J Exp Med, 2007; Sinha P et al. J Immunol, 2007; Jeisy-Scott V et al. PloS One, 2011). This is mainly attributed to their ability to induce IL-10 and reactive oxygen species (J Immunol, 2007; Jeisy-Scott V et al. PloS One, 2011; Cesar A Corzo

et al. J Immunol, 2009). TLR7-KO MDSCs produced IL-10 as well as IL6 in mice lung after S. japonicum infection (Fig 4C). Interestingly, IL6 has been shown to amplify the Th2 response (Tibbitt CAet al. Immunity. 2019). The ability of TLR7-KO MDSCs to coproduce IL-10 and IL6 may be one mechanism that encourages the Th2 bias observed in TLR7-KO mice during S. japonicum infection. It suggested that TLR7 deficiency might increase MDSCs accumulation and Th2 biased response to S. japonicum infection in the lungs of mice, which might be related to pulmonary lesions in infected TLR7-KO mice (eisy-Scott V et al. PloS One, 2011; Leist S R et al. Cell, 2020).

Moreover, the mechanism of immune response induced by schistosoma japonicum is very complex: non-specific immunity and specific immunity are mutually conditional and complementary; humoral and cellular immunity regulate and balance each other; interaction between schistosoma antigens and host MHC; a variety of immune cells, including macrophages, NK cells, B cells and T cells, are involved in the pulmonary lesions in schistosomiasis, and these factors may affect the pathogenesis of schistosomiasis (Zhao Y et al. BMC Infect Dis. 2019; Houlder EL et al. Front Immunol, 2021; Souza COS et al. Front Immunol, 2018). Therefore, in the lungs of infected mice, TLR7-KO may affect the immune response of other immune cells, such as B cells and NK cells, and jointly affect the pulmonary lesions of infected mice (Wei H et al. PLoS Negl Trop Dis, 2021; Dianhui Ch et al. Innate Immun, 2019). This needs to be further studied. We included it in the revised "Discussion".

4. Many descriptions in the background and discussion are unclear, eg, "TLR7 could delay the progression of *S. japonicum* infection-induced lung disease mainly through MDSCs were involved in the TLR7-mediated immune response".

Response: *Thank you. We have followed your suggestion, and made the necessary changes in the revised manuscript. "TLR7 could delay the progression of S. japonicum infection-induced lung disease mainly through MDSCs were involved in the TLR7-mediated immune response" is replaced by "TLR7 signaling inhibits the accumulation and function of MDSCs in S. japonicum infected mouse lung by down-regulating the expression of PD-L1/2 and secreting of IL-10, via NF-κB signaling".*
